# Ethical burdens of COVID-19 testing: the case for a research agenda to improve effectiveness and equity in pandemic response

**DOI:** 10.3389/fpubh.2025.1542587

**Published:** 2025-03-06

**Authors:** Dana Howard, Abigail Norris Turner, Julianna Nemeth, Tasleem J. Padamsee

**Affiliations:** ^1^Department of Biomedical Education and Anatomy, Center for Bioethics, College of Medicine, The Ohio State University Wexner Medical Center, Columbus, OH, United States; ^2^Department of Philosophy, College of Arts and Sciences, The Ohio State University, Columbus, OH, United States; ^3^College of Medicine and College of Public Health, The Ohio State University, Columbus, OH, United States; ^4^College of Public Health, The Ohio State University, Columbus, OH, United States

**Keywords:** COVID-19, ethics, socioeconomic factors, decisional burdens, health equity, research methods

## Abstract

US public health response to COVID-19 has focused on increasing availability and access to viral testing, which raises three sets of potential problems: (1) lack of testing uptake, (2) diminished public health impact of testing, and (3) loss of access to necessary social goods and supports. Moreover, these problems are encountered *differentially* in affluent vs. disadvantaged communities. If not addressed, these problems could exacerbate health disparities via the public health strategies that aim to lower the population-level impact of COVID. These problems also risk undermining trust in public health interventions more broadly and pose challenges to the sustainability of testing programs moving forward. In this perspective essay, we argue that public health research must aim to document and understand the mechanisms through which living in structurally disadvantaged environments exacerbates not only the logistical and material burdens of COVID-19 testing, but also the ethical and social burdens it creates. Such research will facilitate development of targeted interventions that empower people to make the testing-related decisions that best serve both their own interests and those of their broader communities.

## 1 Introduction

The U.S. public health response to COVID-19 has relied heavily on viral testing to suppress outbreaks, monitor case trends, protect vulnerable populations, direct resources, and implement population-level interventions to improve the health of the public (1). Many policy and research efforts have focused on increasing availability and access to COVID-19 testing modalities—including both facility-based and at-home testing—as well as addressing medical mistrust, disparate access, misinformation, and logistical barriers to testing (2–9). While these approaches are important, they fail to capture critical additional factors having to do with the *ethical burdens* many face in relation to COVID-19 testing. Even people who generally endorse testing as a protective public health measure may still face fraught ethical questions about whether or not to test on any particular occasion. In confronting these choices, individuals must not only decide whether their symptoms or potential COVID exposure warrant the use of a testing resource that may be hard to come by; but also must consider the possible impacts a positive result may have on their ability to engage in the normal activities of their lives and to live up to their responsibilities to others close to them.

There has been important discussion of how the success of COVID-19 testing programs depends on the discreet decisions and voluntary actions of individuals who must reckon with specific barriers to testing and the downstream consequences of COVID-19 testing and COVID-19 positivity. The moral dilemmas people face surrounding COVID-19 testing, however, have yet to be explored conceptually or measured empirically. In this essay, we argue that there is an urgent need to fully capture how testing-related choices are structured by barriers and facilitators of COVID-19 testing and post-test prevention behavior, and also by access to social goods and public services. Furthermore, public health research must aim to document and understand the mechanisms through which living in structurally disadvantaged environments exacerbates not only the logistical and material burdens of COVID-19 testing, but also the ethical and social burdens it creates. These findings could facilitate development of targeted social and structural interventions to empower people to make testing-related decisions that best serve both their own interests and those of their broader communities.

## 2 Focusing on testing alone may not maximize public health impact

The U.S. public health response has relied heavily on COVID-19 testing to facilitate a wide range of epidemiological and prevention goals, with a primary focus on expanding availability of COVID-19 testing. Focusing on testing availability as the cornerstone public health strategy raises three sets of potential problems: (1) lack of testing uptake, (2) lack of post-test prevention behaviors, and (3) loss of access to necessary social goods and supports. When they occur, these problems limit the ability of COVID-19 testing to have its intended beneficial *public health impact*. In addition, these problems are encountered *differentially* in affluent vs. disadvantaged communities and populations (8, 10). If not addressed, these problems could result in the unintended negative consequence of exacerbating health disparities via public health strategies that aim to lower the transmission of SARS-CoV-2 and COVID-associated morbidity and mortality at a population level. These three problems also risk undermining trust in public health interventions more broadly, and pose challenges to the sustainability of testing programs moving forward (11–14).

### 2.1 Lack of testing uptake

While COVID-19 testing may be widely available through both at-home test kits and facility-based testing in medical and other institutional settings, it will not have the intended individual- and community-level impact if people do not test when recommended (e.g., when they are exposed, experience symptoms, or have reason to confirm their COVID-19 status before participating in a public activity). We cannot assume that testing availability, testing access, and testing uptake are strongly—or even positively—correlated, and the disconnects between these realities may be particularly pronounced in marginalized populations. For instance, having at-home tests *available* at local pharmacies does not render them *accessible* if the purchase price is too high; free testing sites *available* in a neighborhood may not be *accessible* to everyone if use requires internet access or a social security number or if testing is only available during the workday or there is poor public transportation to reach the site.

Further, *access* to free testing options may not necessarily produce high *uptake* if people do not understand where or when to test, or do not recognize enough benefit to testing—for themselves, for their families, or for their communities. To ensure that testing availability leads to accessibility and expanded uptake, a number of questions must be explored: (1) To what extent does testing availability increase perceived access? (2) Under what conditions does an available, accessible testing resource lead to use? (3) What individual- and community-level considerations account for gaps between availability, access, and uptake? (4) How do these patterns vary across various affluent and disadvantaged communities?

### 2.2 Lack of post-test prevention behaviors

Even when people use COVID-19 testing, it may still not achieve the *intended public health impact* unless they also adopt post-test prevention behaviors. In order for testing to reduce SARS-CoV-2 transmission and subsequent COVID-19 related morbidity and mortality, multiple follow-up actions must be available to, and taken by, individuals who test positive. These include: staying home from work or school; skipping or altering everyday activities such as using public transportation or attending social gatherings; temporarily changing living situations and care responsibilities to protect close others; seeking early treatment [such as nirmatrelvir and ritonavir tablets (Paxlovid™) soon after a positive test] or subsequent healthcare if symptoms become serious; and notifying close contacts about one's positive status (4, 8, 15).

Prioritizing individual testing as the central surveillance and prevention mechanism (instead of, for instance, undertaking widespread wastewater testing combined with more focused individual testing) places these public health burdens onto individuals (14, 16–19). This prioritization can be seen as the result of decades of austerity measures in neoliberal political contexts, which have shifted the responsibility for health and wellbeing from the state to individuals (20–23). Without either the resources or the knowledge of how to implement follow-up behaviors, testing is not only rendered less effective as a public health intervention; it can also place the blame on individuals who may have been acting under a constrained set of choices (24). Further, recommending and reminding individuals to test may lead to mistrust toward governmental entities insofar as people do not feel they have the resources or support to do what official guidance tells them is right (15).

To ensure that expanded uptake of testing leads to its intended public health benefit, a number of further questions must be explored: (1) To what extent is testing associated with post-test prevention behaviors? (2) What reasons and life circumstances underlie the use—and lack of use—of post-test prevention behaviors? (3) To what extent is testing availability (or uptake) associated with lower COVID-19 caseloads or hospitalizations? (4) How do these patterns vary between various affluent and disadvantaged communities?

### 2.3 Loss-of-access consequences and concerns

While testing is intended to reduce SARS-CoV-2 transmission and mitigate the negative impacts of COVID-19, prioritizing policy interventions that focus primarily on testing access and uptake may, even if successful in their aims, have concomitant, unintended negative consequences for public health more generally. People may lose—or fear losing—access to social and public supports that do not allow participation by individuals who have active COVID-19, such as: school or daycare; disability services or eldercare; housing supports such as shelters or group homes; public transportation; social services such as food pantries, case workers, or home aides; routine screenings or other healthcare services unrelated to COVID-19; and appearing in court to resolve legal or family matters. In most cases, economically disadvantaged groups will suffer significantly greater burdens by the loss of these services and supports.

Some loss-of-access consequences have to do with employment, such as lost wages or inability to keep or advance in a job. Some involve formal, enforceable barriers (e.g., showing a negative test to participate), while other barriers may not be formally enforced but still influence individual behaviors (e.g., signs asking people not to ride the bus if they have COVID symptoms). These loss-of-access consequences of a robust testing apparatus are ethically problematic in two ways: first, the fear of loss of access may be a deterrent to testing (25); and second, loss of access could lead to more general negative health and welfare outcomes.

To forecast and address these loss-of-access consequences, researchers must further explore the following questions: (1) To what extent have positive tests been associated with the experience of losing access to social and public supports? (2) To what extent are positive tests associated with the perceived threat of losing access to social and public supports? (3) Are there associations (positive or negative) between “proof of negative test” requirements and testing uptake? (4) How do these patterns vary between various affluent and disadvantaged communities?

These three problems highlight the ways that expanded testing access and even expanded uptake may not result in the intended public health benefit, and how they risk exacerbating existing health disparities and undermining trust in public health authorities and interventions. To prevent these negative outcomes, public health researchers must study the contours of these challenges, including the ways they affect ethical choices and COVID-related prevention behaviors.

## 3 Understanding the ethical aspects of testing choices

In order to comprehensively assess or improve the success of public health testing policies, we must approach testing as a complex social practice and expand our consideration of testing-related barriers beyond logistical, informational, and financial gaps (4). It takes intensive cognitive work and ethical deliberation to weigh the costs and benefits of testing—for oneself and others—and to determine whether and when to test, notify others of one's results, or change one's normal activities. These challenges cannot be addressed purely through education or by correcting misinformation. Rather than characterizing people who face these challenges—or who decide not to test—as “non-compliant” and seeking interventions to effect behavior change, we must shift our theoretical approach. The deliberative tasks people have been expected to undertake during COVID-19 should be understood in part as ethical challenges, requiring acknowledgment and serious investigation in order to be properly addressed (4, 6, 26).

Furthermore, individuals navigate these ethical challenges within the social, economic, and cultural contexts of their lives; implications of these choices may therefore differ substantially by their social location.

A resident of an economically affluent neighborhood, for instance, may experience the lack of local test kit availability during a COVID-19 surge as a challenge that requires work arounds, such as ordering a test online and working remotely until it arrives. A resident of an economically disadvantaged neighborhood two miles away, however, may lack funds to order a test, a secure place to have it delivered, and the ability to work remotely. What is a logistical challenge for the first individual may be a complex ethical challenge for the second: does the risk of potentially exposing co-workers to SARS-CoV-2 transmission outweigh the risk of losing one's job and the ability to pay rent for my family?

When governments, such as in the US or Sweden, focus their public health policy and messaging on the notion that every individual should do their part to stop the spread of the virus, there is an underlying assumption that each individual has a fairly distributed part to play (19). However, not only is it more costly for some people to act according to public health recommendations, but the costs often differ qualitatively in kind. One of the injustices COVID-19 has made vivid—and that should be a focus of study—is that its disparate impacts cannot be measured solely in terms of health and economic outcomes; there are also disparate impacts on who is saddled with morally conflicting choices and who has the resources to be merely inconvenienced.

## 4 Discussion: roadmap for future research on the ethical burdens of testing

In contrast to the robust existing literature focused on a range of challenges and implications associated with COVID-19 vaccination and vaccine mandates, few empirical studies have examined these ethical and social features of COVID-19 testing. A research agenda on the ethical burdens of COVID-19 testing, and their implications for individuals, communities, and disease spread, could usefully be organized around several empirical and methodological priorities. Critically, the ethical challenges people face should be at the center of research, which could either focus exclusively on ethical components of testing- and post-testing decisions or integrate ethical barriers into broader studies of testing-related barriers.

Studies in this space could be organized around the three sets of ethical challenges articulated above—those associated with lack of testing uptake, diminished public health impact of testing, and concerns about loss of access to social supports and public services. Researchers should also devote special attention to the particular types and degrees of ethical burdens experienced by individuals from economically disadvantaged and socially marginalized groups. Structural barriers—related to race, ethnicity, gender, income, wealth, disability, housing and transportation options, environmental safety, and more—accumulate in these contexts and likely intensify experiences and impacts of ethical challenges (27). Researchers should aim to understand how being part of a disadvantaged community, population, or neighborhood exacerbates the ethical and social burdens of COVID-19 testing.

Attending to the ethical dilemmas that individuals face when it comes to COVID-19 testing should not overshadow the larger scale social transformations that must take place to advance public health—such as combatting historical and social disadvantages (e.g., de-industrialization, health system disinvestment) or racist policies (e.g., redlining and anti-immigrant discrimination). Rather, we wish to call attention to the way that ethical burdens which come about due to these structural features of people's decisional predicament can themselves pose an additional barrier to health-related behaviors. Such insights can facilitate development of targeted interventions and material support to empower people to make testing-related decisions that serve their interests, those of their communities, and the broader health of the public. At minimum, such insights can help policymakers develop public messaging related to testing campaigns that takes seriously the ethical burdens people face.

Methodologically, this research agenda should incorporate a mix of approaches, utilizing not only quantitative measures but also qualitative, ethnographic, and ecological/geographic data. On this point we echo recommendations from Bevan et al. (4), who noted that existing testing research is dominated by cross-sectional surveys, limiting researchers' ability to deeply probe the meaning of individual responses or to allow participants to articulate the ethical dilemmas that they face when their public health duties conflict with other duties and needs. It should also incorporate longitudinal designs; the challenges and solutions associated with testing-related decisions evolve through surges in COVID-19 cases, emergence of new variants, policy changes, and other transitions over time. Finally, it should incorporate community- and place-based methods, in order to identify the impacts of locally specific resources and constraints.

Even as effective vaccination and treatments have become widely available, testing remains a critical component of the response to COVID-19 (28). It will continue to be essential as U.S. society navigates the swings between low and surging caseloads linked to annual weather cycles as well as the periodic emergence of new viral variants. Recurring periods of higher COVID-19 prevalence and more infectious or virulent variants are particularly dangerous for economically disadvantaged populations and socially marginalized communities, in which high proportions of people have both elevated exposure to SARS-CoV-2 and increased risk of severe outcomes from COVID-19 (29–31). Furthermore, COVID-19 testing at public health sites is much less available now than at the height of the pandemic, making the U.S. public health strategy even more reliant on individuals to decide when to test (as evidenced by, for instance, the U.S. government's efforts to send free COVID-19 tests to every household), to procure and take their own tests, and to decide what steps to take when they test positive. Understanding these choices as ethically loaded remains highly relevant, especially for protecting the future health of marginalized populations against COVID and other possible diseases.

## Data Availability

The original contributions presented in the study are included in the article/supplementary material, further inquiries can be directed to the corresponding author.

## References

[1] Peeling RW Heymann DL Teo YY Garcia PJ. Diagnostics for COVID-19: moving from pandemic response to control. Lancet Lond Engl. (2022) 399:757–68. 10.1016/S0140-6736(21)02346-1 34942102 PMC8687671

[2] Asabor EN Warren JL Cohen T. Racial/ethnic segregation and access to COVID-19 testing: spatial distribution of COVID-19 testing sites in the four largest highly segregated cities in the United States. Am J Public Health. (2022) 112:518–26. 10.2105/AJPH.2021.306558 35196059 PMC8887160

[3] Bateman LB Schoenberger YMM Hansen B Osborne TN Okoro GC Speights KM. Confronting COVID-19 in under-resourced, African American neighborhoods: a qualitative study examining community member and stakeholders' perceptions. Ethn Health. (2021) 26:49–67. 10.1080/13557858.2021.1873250 33472411 PMC7875151

[4] Bevan I Stage Baxter M Stagg HR Street A. Knowledge, attitudes, and behavior related to COVID-19 testing: a rapid scoping review. Diagn Basel Switz. (2021) 11:1685. 10.3390/diagnostics11091685 34574026 PMC8472251

[5] Kas-Osoka C Moss J Alexander L Davis J Parham I Barre I. African Americans views of COVID-19 contact tracing and testing. Am J Infect Control. (2022) 50:577–80. 10.1016/j.ajic.2022.02.032 35263614 PMC8898856

[6] Lee RM Handunge VL Augenbraun SL Nguyen H Torres CH Ruiz A. Addressing COVID-19 testing inequities among underserved populations in Massachusetts: a rapid qualitative exploration of health center staff, partner, and resident perceptions. Front Public Health. (2022) 10:838544. 10.3389/fpubh.2022.838544 35400042 PMC8987278

[7] Lieberman-Cribbin W Alpert N Flores R Taioli E. Analyzing disparities in COVID-19 testing trends according to risk for COVID-19 severity across New York City. BMC Public Health. (2021) 21:1717. 10.1186/s12889-021-11762-0 34548041 PMC8454292

[8] Jimenez ME Rivera-Núñez Z Crabtree BF Hill D Pellerano MB Devance D. Black and Latinx community perspectives on COVID-19 mitigation behaviors, testing, and vaccines. JAMA Netw Open. (2021) 4:e2117074. 10.1001/jamanetworkopen.2021.17074 34264327 PMC8283554

[9] Nemeth J Padamsee T Writer's Team. Ohio's COVID-19 Populations Needs Assessment: Minimizing the Disparate Impact of the Pandemic and Building Foundations for Health Equity. College of Public Health, The Ohio State University (2020). Available at: https://go.osu.edu/inequitable-burdens-covid-19 (accessed February 25, 2025).

[10] Crozier J Christensen N Li P Stanley G Clark DS Selleck C. Rural, underserved, and minority populations' perceptions of COVID-19 information, testing, and vaccination: report from a southern state. Popul Health Manag. (2021) 25:413–22. 10.1089/pop.2021.0216 34637631

[11] Goldberg DS. Social justice, health inequalities and methodological individualism in us health promotion. Public Health Ethics. (2012) 5:104–15. 10.1093/phe/phs01330866982

[12] Holden T Bourke L. Rural community wellbeing. Rural Soc. (2014) 23:208–15. 10.1080/10371656.2014.11082065

[13] Becker MH. A medical sociologist looks at health promotion. J Health Soc Behav. (1993) 34:1–6. 10.2307/2137300 8463632

[14] Lowenberg JS. Health promotion and the “ideology of choice.” *Public Health Nurs*. (1995) 12:319–23. 10.1111/j.1525-1446.1995.tb00155.x 7479540

[15] Lee SJ Bevan I Street A. The hidden burden of medical testing: public views and experiences of COVID-19 testing as a social and ethical process. 10.2139/ssrn.4031578 36180839 PMC9524338

[16] Byron K Howard D. “Hey everybody, don't get pregnant”: Zika, WHO and an ethical framework for advising. J Med Ethics. (2017) 43:334–8. 10.1136/medethics-2016-103862 27920162

[17] Beck U. Risk Society : Towards a New Modernity. London: Sage Publications (1992).

[18] Foucault M. Lecture 11, 17 March 1976. In: “Society Must Be Defended”: Lectures at the Collège de France 1975–76. 1st Picador pbk., Ed. Picador (2003).

[19] Giritli Nygren K Olofsson A. Managing the COVID-19 pandemic through individual responsibility: the consequences of a world risk society and enhanced ethopolitics. J Risk Res. (2020) 23:1031–5. 10.1080/13669877.2020.1756382

[20] Marelli L Kieslich K Geiger S. COVID-19 and techno-solutionism: responsibilization without contextualization? Crit Public Health. (2022) 32:1–4. 10.1080/09581596.2022.2029192

[21] Hildebrandt T Bode L Ng JSC. Responsibilization and sexual stigma under austerity: surveying public support for government-funded PrEP in England. Sex Res Soc Policy. (2020) 17:643–53. 10.1007/s13178-019-00422-z

[22] Stol YH Schermer MHN Asscher ECA. Omnipresent health checks may result in over-responsibilization. Public Health Ethics. (2017) 10:35–48. 10.1093/phe/phw034

[23] Viens AM. Neo-liberalism, austerity and the political determinants of health. Health Care Anal. (2019) 27:147–52. 10.1007/s10728-019-00377-7 31297702

[24] Bird CE Rieker PP. Gender and Health. New York, NY: Cambridge University Press (2008). 10.1017/CBO9780511807305

[25] Knight KR Duke MR Carey CA Pruss G Garcia CM Lightfoot M. COVID-19 Testing and vaccine acceptability among homeless-experienced adults: qualitative data from two samples. J Gen Intern Med. (2022) 37:823–9. 10.1007/s11606-021-07161-1 34704204 PMC8547296

[26] White RD Wyn J Albanese P. Youth and Society : Exploring the Social Dynamics of Youth Experience, 2nd Edn. Oxford: Oxford University Press (2008).

[27] Bailey ZD Krieger N Agénor M Graves J Linos N Bassett MT. Structural racism and health inequities in the USA: evidence and interventions. Lancet. (2017) 389:1453–63. 10.1016/S0140-6736(17)30569-X 28402827

[28] Breza E Stanford FC Alsan M Alsan B Banerjee A Chandrasekhar AG. Effects of a large-scale social media advertising campaign on holiday travel and COVID-19 infections: a cluster randomized controlled trial. Nat Med. (2021) 27:1622–8. 10.1038/s41591-021-01487-3 34413518 PMC8440209

[29] Singu S Acharya A Challagundla K Byrareddy SN. Impact of social determinants of health on the emerging COVID-19 pandemic in the United States. Front Public Health. (2020) 8:406. 10.3389/fpubh.2020.00406 32793544 PMC7385373

[30] Yearby R. Structural racism and health disparities: reconfiguring the social determinants of health framework to include the root cause. J Law Med Ethics. (2020) 48:518–26. 10.1177/1073110520958876 33021164

[31] Benfer EA Mohapatra S Wiley LF. Health justice strategies to combat the pandemic: eliminating discrimination, poverty, and health disparities during and after COVID-19. Yale J Health Policy Law Ethics. (2020) 19:122–71. 10.2139/ssrn.3636975

